# Supplementary figures and images for: Sodium Thiosulphate-Loaded Liposomes Control Hydrogen Sulphide Release and Retain Its Biological Properties in Hypoxia-like Environment
Source: Antioxidants (Basel). 2022 Oct 24;11(11):2092. doi: 10.3390/antiox11112092 (PMC9686859; doi:10.3390/antiox11112092)

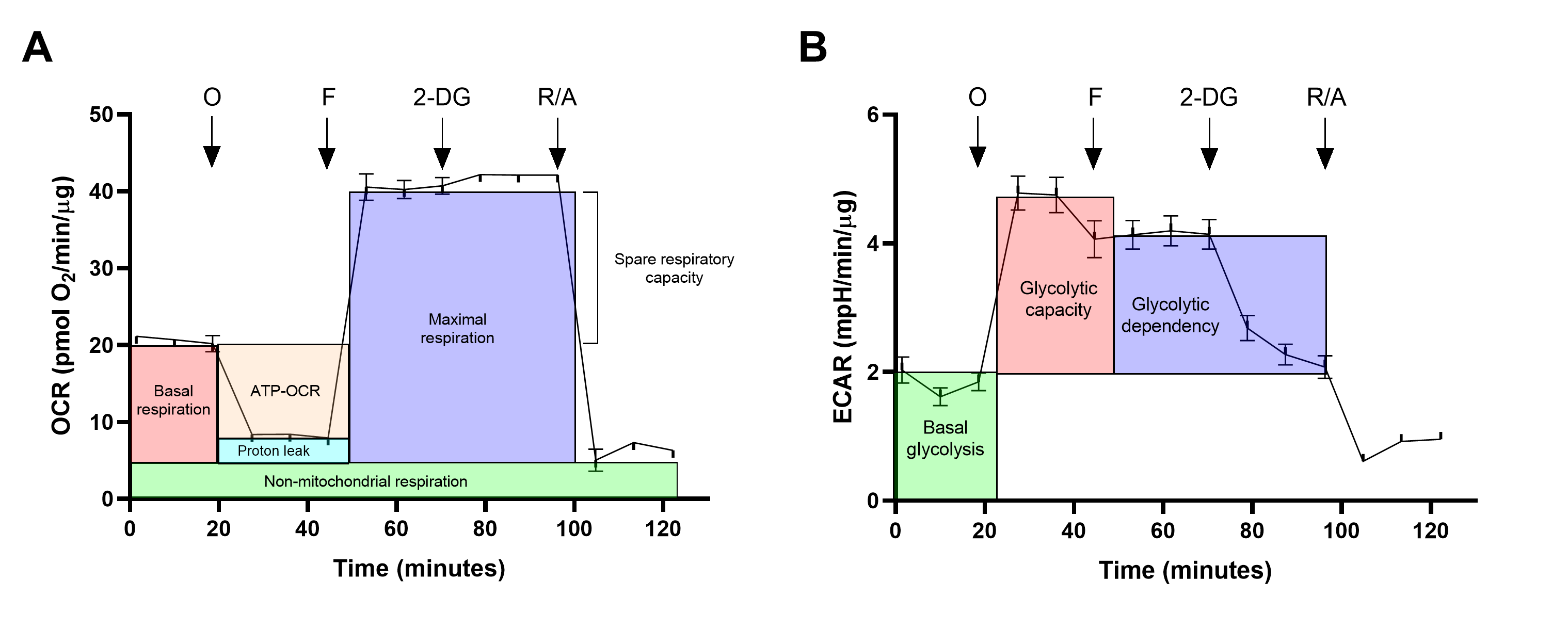

Supplement: Supplementary file 1 [file antioxidants-11-02092-s001.zip › Supplementary Figure S1.tif]

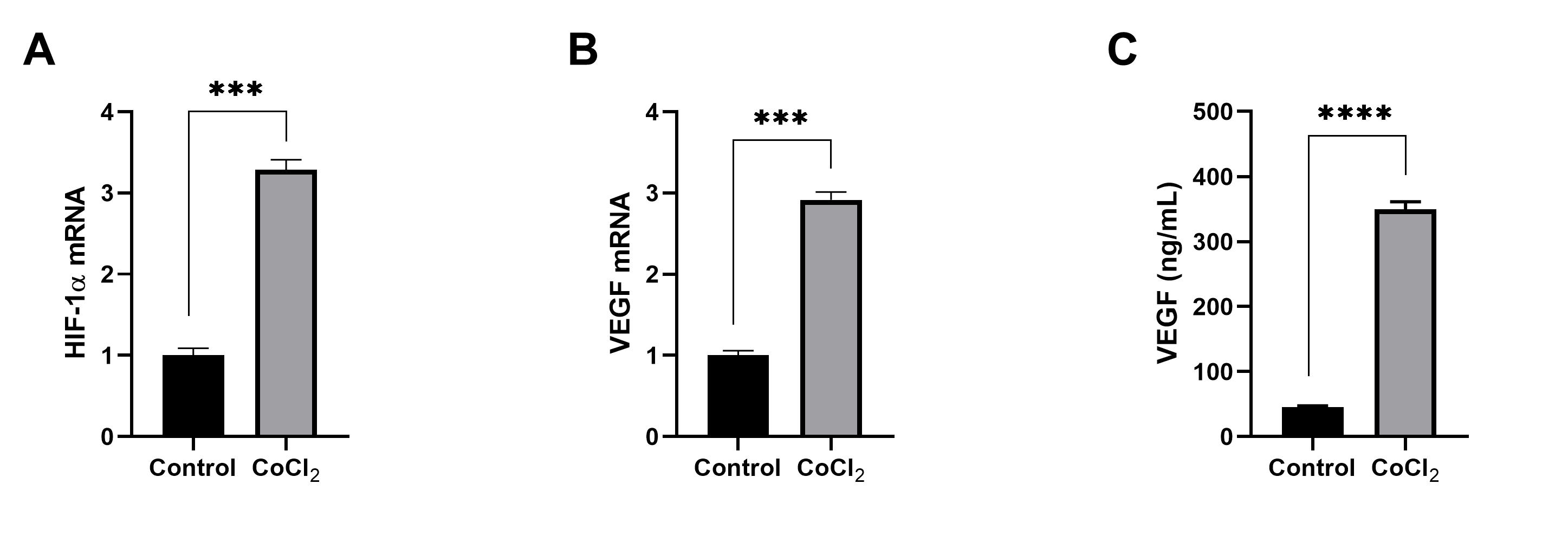

Supplement: Supplementary file 1 [file antioxidants-11-02092-s001.zip › Supplementary Figure S2.tif]
